# Developmental trajectories of schizotypal personality disorder-like behavioural manifestations: a two-year longitudinal prospective study of college students

**DOI:** 10.1186/1471-244X-13-323

**Published:** 2013-11-29

**Authors:** Fu-lei Geng, Ting Xu, Yi Wang, Hai-song Shi, Chao Yan, David L Neumann, David HK Shum, Simon SY Lui, Eric FC Cheung, Raymond CK Chan

**Affiliations:** 1 Neuropsychology and Applied Cognitive Neuroscience Laboratory, Key Laboratory of Mental Health, Institute of Psychology, Chinese Academy of Sciences, 16 Lincui Road, 100101, Chaoyang District, Beijing, China; 2 University of Chinese Academy of Sciences, Beijing, China; 3 Key Laboratory of Behavioral Science, Laboratory for Functional Connectome and Development, Institute of Psychology, Chinese Academy of Sciences, Beijing, China; 4 North China Electric Power University, Beijing, China; 5 School of Psychology and Cognitive Science, East China Normal University, Shanghai, China; 6 Behavioural Basis of Health Research Program, Griffith Health Institute, Griffith University, Gold Coast, Australia; 7 Castle Peak Hospital, Hong Kong Special Administrative Region, Hong Kong, China

**Keywords:** Schizotypal personality disorder, Developmental trajectories, Psychosocial function, Latent class growth analysis

## Abstract

**Background:**

Previous evidence has shown that schizotypal personality disorder (SPD) is part of the schizophrenia spectrum. Few studies have examined latent classes in the developmental trajectories of SPD features over time in individuals with SPD features.

**Methods:**

We adopted a longitudinal prospective study design to follow up a cohort of 660 college students during a two-year period. Participants’ SPD-like symptoms and psychosocial function were measured by a comprehensive set of questionnaires that covered SPD features and cognitive, emotional, and psychosocial functions. Latent class growth analysis was used to examine the trajectory classes.

**Results:**

Three trajectory classes were identified: a low, a medium, and a high SPD features group. Participants in the low group reported few SPD features and their symptoms declined over time. The medium group students had more SPD features than the low group and these symptoms stabilized during the follow up period. Participants in the high group reported the most SPD features and their symptoms increased over time. The three groups differed in paranoid thoughts, psychological distress, neurocognition function, and emotional expression over time. Results of multivariate regression analysis suggested that paranoid thoughts, emotional experience and prospective memory were predictors of social functioning in the high SPD feature group.

**Conclusions:**

Our findings suggest that individuals with SPD features may be delineated into different developmental subgroups and these subgroups differ significantly in psychosocial function. Delusions, emotion, and prospective memory may be important features to consider in early diagnosis and interventions for individuals predisposed to SPD and schizophrenia.

## Background

Schizotypal personality disorder (SPD) is a personality disorder characterized by social and interpersonal deficits, cognitive or perceptual distortions and eccentric behavior. The prevalence of SPD is about 3% in the general population [1]. Individuals with SPD features have been shown to exhibit extensive impairment in multiple domains of psychosocial function, including executive function [2-4], prospective memory [5,6], emotion processing [7-9], social interaction and social functioning [10,11]. Previous studies have also established that SPD lies within the schizophrenia spectrum, with demonstrated genetic and developmental links with schizophrenia [12,13]. However, most of these were cross-sectional studies.

Few longitudinal studies have examined the change of SPD symptoms in adults over time [14-17]. Most of these studies found that SPD symptoms tend to decline over time. For example, Shea et al. followed up 82 clinically diagnosed SPD patients and found that only 34% of them retained the same diagnosis one year later and that the mean number of SPD diagnostic criteria met also declined over time [18]. Lenzenweger et al. evaluated 250 students three times over four years and found that there was a modest decrease in SPD features among them [19]. Gooding et al. were among the first to examine whether psychometrically-defined individuals with SPD would be at an increased risk for psychological disturbance over time [20]. They interviewed 135 at-risk individuals with SPD and followed them up five years later. Compared to the controls, the at-risk groups reported more frequent and severe psychotic-like experiences. Nevertheless, the study only included two time points of assessment and no additional cognitive and psychosocial functions were assessed. As a result, the developmental trajectories of these psychometrically-defined SPD could not be systematically examined. Moreover, empirical studies on psychometrically-defined SPD suggested that two subgroups may be identified among individuals with SPD, that is, one group with stable and moderate to high SPD features and another group with relatively unstable and low to moderate SPD features [21]. It is still not fully known whether changes in SPD features would affect the emotional and psychosocial function of individuals with SPD, both at the clinical and psychometrically-defined levels.

Researchers have only just begun to explore the developmental heterogeneities in the course of personality disorders. Hallquist and Lenzenweger found that there were three latent trajectories in their sample of clinically diagnosed personality disordered individuals and healthy controls [22]. In the personality disorders group, the three latent trajectories were rapid symptom remission, slow symptom decline, and a relative absence of symptoms. In the healthy control group, latent trajectories were characterized by stable, minor symptoms; rapid or gradual remission of subclinical symptoms; or emergence of symptoms of avoidant, obsessive-compulsive or paranoid personality disorders. Clinical and epidemiological studies have also indicated that there might be heterogeneous trajectories in the course of clinical SPD.

Raine proposed that there may be two types of SPD, namely neuro-SPD and pseudo-SPD [13]. The former is associated with a relatively early onset of the features in childhood and shows temporal stability, and finally develops into full-blown psychosis. The latter is characterized by a variable age of onset and shows a greater temporal fluctuation in symptom severity, and these individuals might not eventually develop full-blown psychosis. To date, no empirical data have been collected to test this hypothesis and longitudinal studies are needed to explore the possibility of latent subgroups in the trajectories of either clinical or psychometrically-defined SPD.

The purpose of this study was to explore the emotional and psychosocial functioning of individuals with SPD features over a two-year period. In particular, we attempted to identify latent classes in the trajectories of SPD features. Given the aforementioned studies of SPD [13,21,22], we hypothesized that there would be at least three latent trajectories: high, medium and low level SPD features throughout the two-year period. Moreover, we examined whether the identified latent classes exhibited differential cognitive, emotional and social functioning. Given prior preliminary findings that individuals with SPD features may have poor cognitive and emotional functioning [2-9], we hypothesized that individuals in the latent trajectory with high levels of SPD features would be associated with worse self-reported outcome in terms of cognitive, emotional, and social functioning, whereas individuals in the latent trajectory with low levels of SPD features would be associated with the best outcomes on these measures.

## Methods

### Sample and procedure

Participants were recruited from the North China Electric Power University and Capital Normal University in Beijing, China. Participants were recruited by announcements before a class by a teacher. Those students who were willing to take part in the study stayed behind after class to complete the questionnaires. During 10–13 November 2008, 660 freshmen were assessed by a set of self-administrated questionnaires capturing SPD traits and corresponding to cognitive, emotional, and psychosocial functioning. After the baseline evaluation, participants were followed up and re-evaluated with the same set of questionnaires every six month up to two years (i.e., three times). A total of 594 participants were followed up at the second assessment (time point 2), 504 at the third assessment (time point 3), and 355 at the fourth assessment (time point 4). In addition, we added a scale to measure executive function at time points 2, 3, and 4, and a scale to measure social functioning at time point 4. At time point 1, the mean age of the participants was 19.1 years (*SD* = 0.8) and 52.1% of the participants were male.

The survey was administered in a group format and participants were asked to complete the questionnaires within one hour in the classroom setting during university term time. The surveys were administered by well-trained research assistants in a standard format. Before the evaluation began, the research assistants briefly introduced the purpose of the study and informed consent was obtained from each participant. This procedure for data collection was approved by the Ethics Committee of the Institute of Psychology, Chinese Academy of Sciences. Each participant received 10 RMB for completing the questionnaires each time.

### Measures

#### SPD features

SPD features were assessed using the Schizotypal Personality Questionnaire (SPQ) [23]. It incorporates 74 items rating on a “yes/no” scale, including nine dimensions: ideas of reference, excessive social anxiety, odd beliefs or magical thinking, unusual perceptual experiences, odd or eccentric behavior, absence of friends, odd speech, constricted affect, and suspiciousness or paranoid ideation. These nine symptoms can be reduced to three factors: cognitive-perceptual, interpersonal, and disorganized. The Chinese version of the SPQ was revised by Chen et al. with good reliability and validity [24].

#### Paranoid thoughts

The Paranoia Checklist (PIC) was used to measure participants’ paranoid thoughts [25]. It consists of 15 items with responses made on a 5-point scale for frequency, degree of conviction, and distress, with a possible total score range of 45-225. A higher score indicates greater severity of paranoid thoughts. The present study adopted the Chinese version of the PIC, which has satisfactory psychometric properties [26].

#### Psychological distress

The 28-item General Health Questionnaire was used to assess participants’ psychological distress [27]. It incorporates four dimensions: (1) anxiety and insomnia, (2) depression, (3) somatization, and (4) social dysfunction. Individuals rated each item on a four-point scale ranging from 1 (‘less than usual’) to 4 (‘much more than usual’). The Chinese version GHQ has been found to have excellent psychometric properties [28].

#### Executive function

Participants’ subjective complaints of everyday life executive function were assessed by the Dysexecutive Questionnaire (DEX) [29]. It includes 20 items with responses on a 5-point scale (0 = never and 4 = very often), with a higher score indicating a higher frequency of dysexecutive behaviour. The psychometric properties of the Chinese version of the DEX are satisfactory [30].

#### *Daily memory function*

The Prospective and Retrospective Memory Questionnaire (PRMQ) was used to evaluate the frequency of daily prospective and retrospective memory failures [31]. It contains 16 items rating on a 5-point scale. The total scores range from 16-80, with higher scores indicating poor memory. The validity and reliability of the Chinese version of the PRMQ are satisfactory [5].

#### Emotion experience

The Chinese version Temporal Experience of Pleasure Scale (TEPS) was used to measure individual anticipatory and consummatory pleasurable experience [32]. It includes 20 items on a 6-point Likert scale, from 1 (very false for me) to 6 (very true for me). The questionnaire shows adequate overall reliability with a Cronbach’s alpha of 0.66.

#### Emotional expressivity

The Emotional Expressivity Scale (EES) was used to measure the ability to express emotions [33]. It includes 17 items incorporating two dimensions: suppression and expression. Participants rated each item on a 6-point scale on how they express their emotions and feelings most of the time. The Chinese version of the EES showed high overall internal consistency (Cronbach’s α = 0.82) [34].

#### Social function

Nineteen items selected from the First Episode Social Functioning Scale (FESFS) were used to measure four domains of social functioning: social activities (four items), friend (three items), family (five items), and school (seven items) [35]. Some examples of these items are as follows: “I participate well in extra-curricular group activities such as group sports, organizations, church, and clubs (social activity)”, “I feel I have at least one best friend with whom I can share important things that happen to me (friend)”, “My parents and I typically get along (family)”, “I am able to consistently get good grades (school)”. The participants rated each item on a 4-point Likert scale, from 1 (totally disagree) to 4 (totally agree). A Chinese version of the FESP was adopted [36]. In the present study, the Cronbach’s α of each of the domains on social activities, friend, family, and school were 0.64, 0.58, 0.68, and 0.84 respectively.

### Statistical analysis

Descriptive statistics (means and SD) for SPD symptoms at time point 1, 2, 3 and 4 were examined. Correlation analyses and repeated measures ANOVAs were carried out to examine the stability of schizotypal personality in the whole sample.

Latent class growth analysis (LCGA) was used to identify latent classes in the trajectories of SPD features [37]. LCGA is a kind of mixture modeling, using a categorical latent variable to capture unobserved heterogeneous classes in the development of an outcome over time, with growth parameters presumed to be invariant within classes. In this study, the maximum likelihood estimation in Mplus version 6.1 was employed to estimate the models [38]. The analyses examined models for one through four classes, all with random starting values. To evaluate the model, four fit indices were used, including the Bayesian Information Criterion (BIC), the Akaike’s Information Criterion (AIC), the Lo-Mendell-Rubin Likelihood Ratio Test (LMR-LRT) and entropy. With regard to AIC and BIC, a lower value indicates a better model. The LMR-LRT compares the estimated model with a model with one class less than the estimated model. A low p-value means rejection of the model with one class less. Entropy quantifies classification accuracy, with values close to 1 demonstrating a good separation of classes.

After trajectory classes were identified, one-way ANOVAs were conducted to examine group differences in demographic, paranoid thoughts, psychological distress, executive function, daily memory functioning, emotion experience, emotion expression, and social function. In order to correct for inflated Type I error due to multiple tests of ANOVA, the significant p values were set p < 0.002. Finally, multivariate regression analyses were performed to identify predictors of social function in the high SPD features group.

Apart from latent class growth analyses, all other analyses were performed using SPSS 16.0. When performing LCGA, the maximum likelihood estimation in Mplus version 6.1 was used to deal with missing data with assumption of missing at random. To avoid bias in other analyses, expectation maximization algorithm was applied to impute missing data. In the imputation procedure, we used all longitudinal study variables, depression, gender, and latent class as covariates.

## Results

### Descriptive statistics

Means and SDs for the SPQ total scores over time are presented in Table 1. Repeated measures ANOVA results showed that SPQ total scores declined over time (*F* = 90.30, *df*=3, *p* <0.01). Pearson correlations indicated that schizotypal personality features were moderately stable over the two-year period. The correlation coefficients among the four waves varied from 0.53 to 0.77.

**Table 1:** Correlations, means, and standard deviations for SPQ total scores (n = 660)

	**SPQ time point 1**	**SPQ time point 2**	**SPQ time point 3**	**SPQ time point 4**
Mean	22.78	22.05	18.09	18.06
SD	10.05	11.65	12.49	13.68
SPQ Time Point 1	1.00			
SPQ Time Point 2	0.65	1.00		
SPQ Time Point 3	0.53	0.76	1.00	
SPQ Time Point 4	0.53	0.76	0.77	1.00

*Note:* SPQ indicates Schizotypal Personality Questionnaire. All correlation coefficients are significant at p < 0.01.

### The trajectory classes of schizotypal personality symptoms

Table 2 presents AIC, BIC, entropy, and LMR-LRT results of different classes. The statistics showed that the three-class model was the best one. Between a two-and a three-class model, AIC and BIC decreased significantly and the entropy increased. The four-class model was not well supported. Moving from a three-class to four-class model, the decrease in AIC and BIC were small, the entropy was lower, and a non-significant LMR-LRT result did not support an additional class (*p* = 0.301).

**Table 2:** Criteria for deciding the number of classes in the study

**No. of classes**	**AIC**	**BIC**	**Entropy**	**LMR-LRT**
2	15,740	15,790	0.782	0.0000
3	15,557	15,624	0.811	0.0059
4	15,518	15,603	0.750	0.3010

*Note:* AIC = Akaike’s Information Criterion; BIC = Bayesian Information Criterion; LMR-LRT = the Lo-Mendell-Rubin Likelihood Ratio Test.

**Figure 1:**
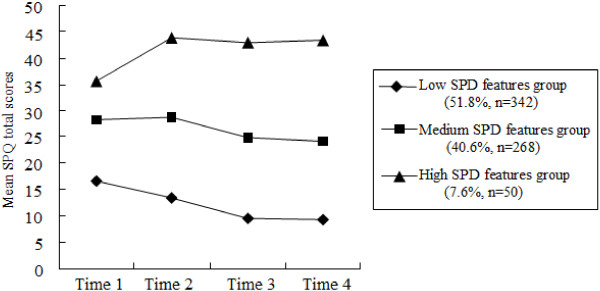
Mean schizotypal personality questionnaire (SPQ) total scores across four time points for the low SPD features group, medium SPD features group, and high SPD feature group.

SPQ total scores for the preferred three-class trajectory are displayed in Figure 1. The first group, low SPD features group (51.8% of the sample) declined over time (intercept = 16.84, *p* <0.01; slope = -4.26, *p* <0.01; quadric = 0.54, *p* <0.01). The second group, moderate SPD features group (40.6% of the sample) was stable over time (intercept = 28.46, *p* <0.01; slope = -0.35, *p* =0.77; quadric = -0.52, *p* =0.13). The third group, high SPD features group, increased over time (intercept = 35.07, *p* <0.01; slope = 8.58, *p* <0.01; quadric = -2.17, *p* <0.01).

### Comparison by trajectory class in demographic and psychosocial factors

Chi-square test showed that gender was significantly associated with class (*χ*^2^ = 6.31, *df* = 2, *p* = 0.043). The percentage of females in the low SPD features group, the moderate SPD features group, and the high SPD-features group were 51.2%, 42.2% and 56.0% respectively. One way ANOVA showed no group difference in age (*F* = 2.59, *df*=2, *p* = 0.076).

As shown in Table 3, the three groups differed in paranoia checklist total scores (*F*_*time1*_ = 72.16, *F*_*time2*_ = 83.82, *F*_*time3*_ = 115.03, *F*_*time4*_ = 76.64, all *df*=2, *p* < 0.001), GHQ total scores (*F*_*time1*_ = 78.38, *F*_*time2*_ = 65.70, *F*_*time3*_ = 97.64, *F*_*time4*_ = 67.49, all *df*=2, *p* < 0.001), PRMQ total scores (*F*_*time1*_ = 36.73, *F*_*time2*_ = 34.48, *F*_*time3*_ = 50.71, *F*_*time4*_ = 17.58, all *df*=2, *p* < 0.001), EES total scores (*F*_*time1*_ = 7.24, *p* = 0.001; *F*_*time2*_ = 6.11, *p* = 0.002; *F*_*time3*_ = 8.22, *p* < 0.001; *F*_*time4*_ = 4.66, *p* = 0.009; all *df*=2), and DEX total scores(*F*_*time2*_ = 70.00, *F*_*time3*_ = 117.76, *F*_*time4*_ = 111.25, all *df*=2, *p* < 0.001). Bonferroni post-hoc contrasts showed a severity-related pattern in paranoid thoughts, psychological distress and executive function across the three classes. There was no difference between the high SPD-features group and the moderate SPD features group in prospective memory and EES total scores, although they both scored higher than the low SPD features group. The three groups showed no difference in the TEPS total scores at time point 1 (*F* = 0.83, *df*=2, *p* = 0.44), time point 2 (*F* = 1.20, *df=*2, *p* = 0.30), time point 3 (*F* = 0.11, *df*=2, *p* = 0.90), and time point 4 (*F* = 2.15, *df=*2, *p* = 0.12).

**Table 3:** Group comparison on psychological function at four time points (n = 660)

	**Time point 1**	**F**	**Time point 2**	**F**	**Time point 3**	**F**	**Time point 4**	**F**
	**Mean (SD)**		**Mean (SD)**		**Mean (SD)**		**Mean (SD)**	
PIC total scores		72.16		83.82		115.03		76.64
Low group	68.25 (15.58)^a^		68.24 (17.93)^a^		64.70 (16.36)^a^		67.92 (17.67)^a^	
Medium group	84.66 (20.80)^b^		84.76 (22.27)^b^		83.44 (21.92)^b^		83.97 (21.14)^b^	
High group	89.20 (22.93)^b^		100.44 (25.35)^c^		101.54 (28.19)^c^		97.42 (28.54)^c^	
Total	76.50 (20.36)		77.39 (22.83)		75.10 (23.02)		76.67 (22.30)	
GHQ total scores		78.38		65.70		97.64		67.49
Low group	13.77 (6.60)^a^		16.35 (7.83)^a^		15.29 (7.74)^a^		17.05 (8.47)^a^	
Medium group	19.58 (9.04)^b^		22.27 (9.59)^b^		21.97 (8.76)^b^		22.48 (8.71)^b^	
High group	26.76 (10.70)^c^		29.38 (11.00)^c^		31.70 (14.23)^c^		31.02 (11.99)^c^	
Total	17.11 (8.92)		19.74 (9.67)		19.24 (10.00)		20.31 (9.73)	
PRMQ total scores		36.73		34.48		50.71		17.58
Low group	35.99 (8.94)^a^		36.75 (9.23)^a^		35.23 (9.01)^a^		36.56 (8.55)^a^	
Medium group	41.74 (9.72)^b^		42.58 (9.35)^b^		41.08 (8.47)^b^		39.70 (7.65)^b^	
High group	44.02 (9.64)^b^		43.86 (10.61)^b^		45.46 (9.10)^c^		42.29 (8.24)^b^	
Total	38.93 (9.81)		39.66 (9.85)		38.38 (9.44)		38.27 (8.38)	
EES total scores		7.24		6.11		8.22		4.66
Low group	63.76 (10.64)^a^		63.43 (9.67)^a^		64.29 (8.38)^a^		63.83 (7.12)^a^	
Medium group	60.93 (11.25)^b^		61.32 (9.95)^b^		61.75 (9.46)^b^		62.06 (7.83)^b^	
High group	59.14 (11.22)^b^		59.03 (12.40)^b^		60.38 (10.22)^b^		61.96 (8.20)^b^	
Total	62.26 (11.04)		62.24 (10.09)		62.96 (9.07)		62.97 (7.55)	
TEPS total scores		0.83		1.20		0.11		2.15
Low group	79.28 (12.58)		81.77 (12.31)		80.03 (13.38)		79.03 (12.28)	
Medium group	80.59 (13.30)		80.21 (13.24)		79.91 (13.60)		78.54 (13.56)	
High group	80.52 (13.01)		80.47 (12.02)		79.09 (12.58)		74.94 (14.92)	
Total	79.90 (12.91)		81.04 (12.68)		79.91 (13.39)		78.52 (13.05)	
DEX total scores				70.00		117.76		111.25
Low group	NA		20.95 (8.76)^a^		17.29 (9.46)^a^		18.53 (9.52)^a^	
Medium group	NA		27.86 (8.26)^b^		26.10 (8.42)^b^		27.03 (8.08)^b^	
High group	NA		31.85 (7.42)^c^		33.47 (7.39)^c^		34.43 (8.94)^c^	
Total	NA		24.58 (9.31)		22.09 (10.36)		23.19 (10.30)	

*Note:* Different superscript letters (a, b, c) refer to significant differences of mean scores between groups. PIC = Paranoia Checklist; GHQ = General Health Questionnaire; PRMQ = Prospective and Retrospective Memory Questionnaire; EES = Emotional Expressivity Scale; TEPS = Temporal Experience of Pleasure Scale; DEX = Dysexecutive Questionnaire. NA, not applied.

Across the three groups, there were significant differences in the four domains on social function (*F* = 6.46 for social activity, *F* = 9.93 for friend, *F* = 8.71 for family, *F* = 21.83 for school; all *df=*2, *p* <0.01and total social function, *F* = 19.22, *p* <0.01). Posthoc tests (Bonferroni) showed that students in the low SPD features group scored significantly higher in social activity, friend, and family domains than the moderate SPD features group and high SPD features group (see, Table 4). A severity-related pattern was found again in school activity and total social function.

**Table 4:** Group comparisons on social function (n = 660)

	**Low group (LG)**	**Medium group (MG)**	**High group (HG)**	**F**	**Post Hoc**
Social activity	12.00 (1.15)	11.72 (1.05)	11.58 (1.05)	6.46^**^	LG > MG, HG
Friend	9.18 (1.02)	8.90 (0.96)	8.64 (1.03)	9.93^**^	LG > MG, HG
Family	15.65 (1.50)	15.29 (1.40)	14.87 (1.49)	8.71^**^	LG > MG, HG
School	21.74 (2.04)	21.11 (1.78)	19.97 (2.02)	21.83^**^	LG > MG > HG
Total social function	54.95 (4.54)	56.96 (4.27)	58.66 (4.98)	19.22^**^	LG > MG > HG

*Note:* **, p < 0.01.

### Predictors of social function in high SPD features group

Multivariate regression results indicated that emotional experience significantly predicted social function among the high SPD features group students (β_time1_ = 0.53, *p* < 0.01; β_time2_ = 0.55, *p* < 0.01; β_time3_ = 0.26, *p* < 0.05). Emotion expression (β_time2_ = -0.28, *p* < 0.05), paranoid thoughts (β_time3_ = -0.37, *p* < 0.05), and prospective memory (β_time3_ = 0.47, *p* < 0.01) were also significantly associated with social function. Gender, psychological distress and executive function did not predict social function in any regression analysis (see, Table 5).

**Table 5:** Multivariate regression analyses predicting social function in the high group (n = 50)

	**Time point 1**		**Time point 2**		**Time point 3**	
	**β**	**t**	**β**	**t**	**β**	**t**
Gender	-0.05	-0.32	-0.15	-1.10	-0.22	-1.69
Paranoia thoughts	0.06	0.43	0.11	0.75	-0.37	-2.67^*^
Psychological distress	0.01	0.07	0.06	0.34	0.11	0.78
Emotional expression	-0.17	-1.22	-0.28	-2.15^*^	-0.16	-1.28
Emotional experience	0.53	3.78^**^	0.55	3.65^**^	0.26	2.07^*^
Prospective memory	-0.18	-1.28	-0.20	-1.47	-0.47	-3.64^**^
Executive function	NA	NA	-0.12	-0.94	0.16	1.17

*Note:* Gender, 0 = female, 1 = male. *, p < 0.05; **, p < 0.01.

## Discussion

In the present study, we assessed the 660 college students four times over a two-year period. Using LCGA, we found three heterogeneous developmental trajectories (high, moderate, and low SPD features groups) in SPD features. To our knowledge, few studies have examined subgroups in the course of SPD features. Furthermore, ANOVA results indicated that the three classes were significantly different in paranoid thoughts, psychological distress, prospective memory, executive function, emotion expression and social function. Interestingly, in the high SPD features group, paranoid thoughts, emotional experience, emotional expression and prospective memory were significant predictors of social functioning.

As previous studies have shown [18,39], our overall mean-level analysis results found that schizotypal personality symptoms declined over time. The decrease in SPD features in our study may be related to the timing of participant recruitment which coincided with the beginning of college life when participants may encounter adjustment problems. With time, these adjustment problems were expected to decrease which might have led to the observed decrease in SPD features. Many studies have demonstrated that stressful life events could increase individual SPD symptoms [40,41].

In a recent study, Hallquist and Lenzenweger reported for the first time longitudinal heterogeneity of SPD symptoms in patients with personality disorders and healthy controls [22]. They identified two subgroups in the personality disorders group. In the first group, individuals experienced minimal SPD symptoms at baseline that declined significantly over time, with all individuals reporting zero symptoms at the two follow-up assessments. The second group reported subclinical to clinical levels of SPD features at baseline and these symptoms declined significantly over time. No heterogeneous trajectories of SPD symptoms were found in healthy controls. However, they used the mean number of DSM-IV diagnostic criteria of SPD as symptom indicators. Most participants reported very low levels of SPD symptoms, especially among the healthy controls. In addition, the sample size in their study was small. Our findings showed that there may be three classes of developmental trajectories of SPD symptoms. The high SPD features group, which accounted for 7.6% of the sample, deteriorated slightly during the two years. The moderate SPD features group accounted for 40.6% the sample and their SPD features were stable over the two-year period. The low SPD features group accounted for 51.8% of the sample and their SPD features declined over time. It is interesting to note that in the present study the statistical distribution of the three trajectory groups approximated a half-normal distribution, which is common in multi-factorial diseases. Furthermore, the three patterns of change in the study supported the proneness-persistence-impairment model [42]. Contrary to our expectation, we did not find the two types of SPD (neuro-SPD and pseudo-SPD) proposed by Raine [13]. This difference may have been due to the relatively small sample size, relatively short follow-up period, and the relative homogeneity of college students.

In the present study, gender was found to significantly associate with the three latent classes and females were more likely to be in the high SPD features group. Although some studies found males to be more likely to report SPD symptoms [43], prior results regarding the relationship between gender and SPD have been inconsistent. For example, in an epidemiological study of personality disorders, Coid et al. demonstrated that the prevalence of SPD was higher in females than in males [44].

A number of epidemiological studies have reported high prevalence of delusions in non-clinical samples [25,26]. Consistent with previous studies, we found that the three latent classes showed a severity-related pattern in paranoid thoughts. Specifically, participants in the high SPD features group reported more delusion-like experiences than the other two groups. Further, those in the moderate SPD features group reported more delusion-like experiences than the low SPD features group. From the perspective of the psychosis continuum, individuals who report high levels of delusion-like behavoiur may be more likely to develop clinical psychosis [42,45]. More longitudinal studies are needed to further explore the relationship between SPD and psychosis development.

Previous studies have reported a high prevalence of comorbid DSM axis I disorders and SPD [46,47]. For example, in a national epidemiological survey, Pulay et al. found that the prevalence of SPD were 10.7% to 33.1% among respondents with any mood disorder or anxiety disorder [48]. In our study, high SPD features group reported more psychological distress than the low and moderate SPD features groups. Furthermore, the changes in SPD features and psychological distress over time were similar among the three groups. The relationship between SPD and psychological distress is complex. On the one hand, common genetic and environmental factors could contribute to both of them but on the other hand, SPD and affective disorders may share a bidirectional relationship over time. Further research is needed to understand the high comorbidity rate between SPD and DSM axis I disorders.

Another important finding of our study is the different profiles of cognitive, emotional, and social functioning of the identified developmental trajectories of SPD features. A number of cross-sectional studies have reported executive function and memory deficits in individuals with SPD features [2-6]. In this study, we found that participants in the high SPD features group reported significantly more executive dysfunctions than participants in the other two SPD feature groups. Moreover, participants in the moderate and high SPD features groups reported more prospective memory deficits than participants in the low SPD features group. Previous studies have demonstrated that the neural basis of executive function and prospective memory is located mainly in the prefrontal cortices [49]. Meanwhile, recent imaging studies have suggested that prefrontal anatomical abnormalities are common in both patients with schizophrenia and individuals with SPD features [50-52]. Imaging data are needed to examine the relationships between prefrontal cortices and deficits of executive function and prospective memory in SPD.

Emotional processing deficits have also been reported extensively in patients with schizophrenia [8,53]. In a recent study, Phillips and Seidman reviewed emotion processing in individuals at risk for schizophrenia and they found reduced self-reported anhedonia and increased negative affect in the at-risk groups [7]. In the present study, participants in the high and moderate SPD features groups were more likely to suppress their emotions than participants in the low SPD features group. However, there was no difference in hedonic experience among these three groups. Using experience-sampling methods, Myin-Germeys et al. found first-degree relatives of patients with schizophrenia and healthy controls reported similar experience of positive and negative emotions [54]. The negative results of anhedonia in our study may be due to our sample consisting mainly of relatively high-functioning college students, and that emotion processing deficits may arise at a relatively later stage in the course of schizophrenia spectrum disorders.

Impairment in psychosocial functioning is a core criterion for the diagnosis of personality disorders. Although a number of studies have reported social functioning impairments in patients with SPD [11,40], few of these studies examined the risk factors for these impairments. In our study, the three groups differed significantly in social functioning. Further, we found that previous paranoid thoughts, anhedonia, emotional suppression, and subjective complaints of poor prospective memory significantly predicted social function decline in the high SPD features group. This highlights the importance of emotion and prospective memory in the early identification and intervention of high risk groups.

The present study has a number of limitations. First, self-administrated questionnaires were used to measure participants’ SPD features, cognitive, emotional, and social functioning. These subjective reports might have been limited by estimation bias and should be interpreted with caution. A larger representative sample and longer-term follow up are needed to replicate the current present findings. Second, in this study, we did not assess any potential conversion rate from SPD to schizophrenia. Future studies should investigate this issue to identify the incidence and associated risk factors. Third, the results reported in this study were based on psychometrically-defined SPD participants, which may not be generalizable to clinically diagnosed SPD individuals. However, recent studies reported that individuals with psychometrically-defined SPD have been shown to demonstrate impairments at both neuroanatomical and behavioural levels when compared with patients with schizophrenia and healthy controls [6,52,55]. These findings are consistent with Raine’s view that although psychometrically-defined SPD may have a weaker genetic and neurobiological basis compared with clinically diagnosed SPD, psychometrically-defined SPD is postulated to mimic the clinical features of clinically diagnosed SPD and is no less debilitating than the clinical group. The main difference is that these psychometrically-defined SPD individuals may have a somewhat different aetiology, involving more cognitive-emotional and psychosocial influences than the clinically diagnosed SPD individuals. Fourth, we did not recruit any genetically or biologically at-risk individuals with SPD features in our study. Future study should extend the sample inclusion to non-psychotic first-degree relatives of patients with schizophrenia and to examine whether there are any potential differences between “behaviourally at-risk individuals” and “biologically at-risk individuals”. Finally, no imaging data were collected in our study. This, therefore, did not allow us to examine the relationship between structural and functional abnormality among the 3 SPD features group indentified in this study.

## Conclusions

Despite these limitations, our study is one of the few to examine the developmental trajectories of individuals with SPD features and to examine the accompanying changes of cognitive-emotional and psychosocial functioning. We have adopted a LCGA technique, which is a rigorous statistical method that can capture unobserved heterogeneous latent classes of SPD in the development of the cognitive-emotional and psychosocial outcome over a two-year time period. Our findings suggest there were different subgroups of individuals (viz., high, moderate and low SPD features group) with different developmental trajectories of SPD features. The high SPD features group was associated with the worst outcome over a two-year time period. These findings may provide useful information for the early detection and intervention for individuals prone to SPD and schizophrenia.

## Abbreviations

AIC: Akaike’s Information Criterion; ANOVA: Analysis of variance; BIC: Bayesian Information Criterion; DEX: Dysexecutive questionnaire; EES: Emotional expressivity scale; FESFS: First episode social functioning Scale; GHQ: General Health Questionnaire; LCGA: Latent class growth analysis; LMR-LRT: Lo-Mendell-Rubin Likelihood Ratio Test; PIC: Paranoia Checklist; PRMQ: Prospective and Retrospective Memory Questionnaire; RMB: Renminbi; SD: Standard deviation; SPD: Schizotypal personality disorder; SPQ: Schizotypal Personality Questionnaire; TEPS: Temporal Experience of Pleasure Scale.

## Competing interests

The authors declare that they have no competing interests.

## Authors’ contributions

FLG analyzed the data and wrote up the first draft of the manuscript. TX, YW, HSS, CY collected the data, helped analyze the data and assist in writing up the draft. DLN, DHKS, SSYL, EFCC contributed significantly to the writing up of the first draft of the manuscript. RCKC designed the study, interpreted the data and wrote up the first draft of the manuscript. All authors read and approved the final version of the manuscript.

## Pre-publication history

The pre-publication history for this paper can be accessed here:

http://www.biomedcentral.com/1471-244X/13/323/prepub
